# Multidimensional analyses to assess the relations between treatment choices by physicians and patients’ characteristics: the example of COPD

**DOI:** 10.1186/1471-2466-12-39

**Published:** 2012-08-06

**Authors:** Nicolas Roche, Christos Chouaid, Bertrand Delclaux, Yan Martinat, Jean-Michel Marcos, Hervé Pégliasco, Bruno Scherrer

**Affiliations:** 1 Respiratory and Intensive Care Medicine, Hôtel-Dieu Hospital, AP-HP, University Paris Descartes, Paris, France; 2 Respiratory Medicine, Hôpital Saint-Antoine, AP-HP, University Pierre et Marie Curie, Paris, France; 3 Respiratory Medicine, Troyes Hospital, Troyes, France; 4 Parrot Medical Centre, Lyon, France; 5 Respiratory Medicine, Libourne Hospital, Libourne, France; 6 Respiratory Medicine, Clinique Ambroise Paré, Marseille, France; 7 Bruno Scherrer Conseil, Saint-Arnoult en Yvelines, France

**Keywords:** COPD, Factor analysis, Phenotype, Treatment, Management, Guidelines

## Abstract

**Background:**

In some situations, practice guidelines do not provide firm evidence-based guidance regarding COPD treatment choices, especially when large trials have failed to identify subgroups of particularly good or poor responders to available medications.

**Methods:**

This observational cross-sectional study explored the yield of four types of multidimensional analyses to assess the associations between the clinical characteristics of COPD patients and pharmacological and non-pharmacological treatments prescribed by lung specialists in a real-life context.

**Results:**

Altogether, 2494 patients were recruited by 515 respiratory physicians. Multiple correspondence analysis and hierarchical clustering identified 6 clinical subtypes and 6 treatment subgroups*.* Strong bi-directional associations were found between clinical subtypes and treatment subgroups in multivariate logistic regression. However, although the overall frequency of prescriptions varied from one clinical subtype to the other for all types of pharmacological treatments, clinical subtypes were not associated with specific prescription profiles. When canonical analysis of redundancy was used, the proportion of variation in pharmacological treatments that was explained by clinical characteristics remained modest: 6.23%. This proportion was greater (14.29%) for non-pharmacological components of care.

**Conclusion:**

This study shows that, although pharmacological treatments of COPD are quantitatively very well related to patients’ clinical characteristics, there is no particular patient profile that could be qualitatively associated to prescriptions. This underlines uncertainties perceived by physicians for differentiating the respective effects of available pharmacological treatments. The methodology applied here is useful to identify areas of uncertainty requiring further research and/or guideline clarification.

## Background

In several chronic diseases, guidelines remain quite vague on treatments hierarchy, especially when large trials have failed to identify subgroups of particularly good or poor responders to available medications. Recognizing areas of uncertainty could help focusing future research and clarifying guidelines.

COPD is a major cause of disability and premature death worldwide [1]. Smoking cessation is the only way of modifying the natural history of the disease, while other pharmacological and non-pharmacological interventions have the potential for reducing its burden in terms of dyspnea, exercise performance, exacerbations and quality of life [1-5]. Recently, large long-term trials also provided somehow encouraging (although not totally conclusive) results on the possible effects of fixed combinations (namely, salmeterol + fluticasone), long-acting beta2 agonists (LABA, namely salmeterol) and long-acting antimuscarinic agents (LAMA, namely tiotropium) on mortality and decline in lung function [2,3,6,7]. In parallel, during the last decade many studies underlined the multi-component character of the disease [8] and rehabilitation was found highly effective at fighting the decrease in exercise performance and daily activity at all disease stages [9].

But the complexity of the disease implies that there is no unique phenotype: for instance, the weight of comorbidities or the frequency of exacerbations vary from one patient to another [8,10,11], as well as the respective contributions of airways disease and emphysema [12]. This area has been the topic of several recent studies aiming at identifying clinically relevant phenotypes or developing prognostic scores [13,14]. The use of some clinical characteristics has been advocated to guide treatment choices in COPD: FEV_1_ is the main cited criterion, together with the repetition of exacerbations and the level of dyspnea [1]. Some algorithms propose rules to modulate treatments when the disease severity and impact increase [15]. However, while LAMA, LABA or ICS + LABA combinations have been shown to relieve dyspnea, decrease exacerbations frequency and improve quality of life [2,3], studies have been unable to clearly identify subgroups of subjects responding particularly well to a given medication [16-20]. For instance, in one of the above-mentioned clinical trials, a favourable effect of tiotropium on lung function decline was found only in GOLD 2 patients, suggesting the possibility of a better responding subgroup; however, there was no interaction between symptomatic effects and patients’ characteristics [2]. Thus, in many situations several therapeutic options are available, without clear-cut differentiation in terms of target populations.

Finally, several studies including some performed in France found that treatment choices by physicians differ sometimes markedly from guidelines recommendations, e.g., regarding the use of inhaled corticosteroids [21-23]. For these treatments as well as for choices between bronchodilators, the criteria on which physicians rely are quite unclear.

This observational study was performed to identify the patients characteristics associated with treatment choices in COPD patients visiting a respiratory physician at stable state. More generally, it aimed at exploring whether multidimensional analyses performed with no *a priori* hypothesis could link some typologies of clinical characteristics to some typologies of treatments, using data that are readily available in routine practice.

## Methods

### Study design

This was an observational study of COPD patients recruited by 515 respiratory physicians. Each of them was asked to recruit the first 5 consecutive patients with COPD (GOLD diagnostic criteria) who visited him/her at stable state and agreed to participate. All GOLD stages could be enrolled, as well as at-risk patients (*i.e.,* presence of chronic bronchitis with normal lung function, previously GOLD stage 0). Patients were not included if they reported an exacerbation during the last 4 weeks, when no lung function data was available during the past 2 years or when they suffered from a significant comorbidity compromising the short-term prognosis. All patients provided informed consent before inclusion and the study was approved by the ethics committee of Versailles (France).

### Collected data

The following data were recorded: socio-demographic characteristics, risk factors (active and passive smoking, occupational exposures), history of COPD (year of first symptoms and year of diagnosis), comorbidities, number and severity of exacerbations, most recent lung function data, symptoms (cough, sputum production, MRC dyspnea grade) using questions derived from the European Communicty Respiratory Health Survey [24], usual maintenance pharmacological and non-pharmacological treatments, yearly number of visits to GPs and chest physicians, investigations performed during the previous year. Exacerbations were defined as an increase in cough and/or sputum production and/or dyspnea during at least 48 hours and requiring a change in treatment (*i.e.,* at least an increase in the dose of bronchodilators). Their severity was classified as mild (self-managed change in treatment), moderate (requiring a visit to a physician), severe (hospitalisation in a medical ward) or very severe (hospitalisation in an intensive care unit).

### Statistical analysis

The planned sample size was set to 2400 patients to obtain a 2% precision in the prevalence estimate of a patient characteristic that is present in about 50% of the population. A two-step process was followed, first identifying typologies of patients and treatments, then exploring their relationships.

Two analyses were used to find typologies of patients on the one hand and typologies of treatments on the other: the multiple correspondence analysis (MCA) and the ascending hierarchical complete linkage clustering analysis (AHCLCA)[25,26]. MCA allows mapping patients’ characteristics or treatments in a reduced space according to their deviation from independence (association or mutual exclusion). Clustering is based on the distance between variables, which is equal to 1 minus the correlation. This method forces the inclusion of all variables in a given group. As a consequence, small groups are constituted of positively related variables, while larger groups may pool together variables that may be unrelated to each other or even negatively related. Results of MCA and AHCLCA were combined to find a typology.

Once groups of patient characteristics and groups of treatments were defined, multiple logistic regression was used to assess (i) associations between each clinical subtype and the 6 treatment subgroups and (ii) associations between each treatment subgroup and the 6 clinical subtypes, to identify the clinical subtype to which a given treatment type is preferentially prescribed or in which it is underprescribed. These two approaches should provide convergent but not exactly similar results because the odds ratios measuring associations between clinical subtypes and treatment subgroups are not adjusted on the same set of covariates (adjustment on other treatment subgroups in one case and on other clinical subtypes in the other). Canonical analysis of correspondence (CAC)[25,26] and more precisely principal component analysis (PCA) with respect to instrumental variables [27] was then used to map treatment variables and patient characteristics in a reduced space and identify (without any clustering) which patient characteristics are associated to which treatments. Two canonical redundancy analyses were performed: the first one with prescribed COPD medication including oxygen therapy as response variables (16 variables), the second with other (i.e., non-pharmacological) COPD treatments and patient follow-up variables (8 variables: number of antibiotic courses, homeopathy, flu vaccine, pneumococcal vaccine, chest physiotherapy, frequency of visits to respiratory physician, frequency of visits to general practitioners and frequency of lung function tests).

More details on these methods can be found in the electronic supplementary material.

## Results

### Population and treatments characteristics

About 3000 respiratory physicians (*i.e.,* all French respiratory physicians) were contacted; 515 accepted to participate and were compared to a random sample of 245 non-participating physicians: no significant difference was found in terms of type of clinical practice (general hospital, university hospital, private practice). The 515 participating physicians were harmoniously distributed on the whole French mainland territory. They recruited 2494 patients, among whom recent lung function data was unavailable in only 10 (protocol deviations). The general characteristics and treatments of the population are summarized in Table 1.

**Table 1:** Patients, investigations, follow-up and treatments characteristics

**N = 2494**	**Mean ± SD or%**
Age (years)	67.0 ± 10.6
Male (%)	78%
Body mass index (kg/m^2^)	25.84 ± 5.17
Smokers / ex-smokers	25.6% / 62.4%
Chronic bronchitis without airflow obstruction / COPD	23.7% / 76.3%
(GOLD stage): 1 / 2 / 3 / 4	2.9% / 29.7% / 26.1% / 17.6% /
FEV_1_ (L/sec)	53.1 ± 19.6
Duration of chronic bronchitis / COPD (years)	8.0 ± 7.8
Chronic cough / sputum production	70.5% / 72.2%
Chronic cough with sputum production by GOLD stage in COPD patients: GOLD 1/2/3/4	62.0% / 71.9% / 72.4% / 76.4%
Modified Medical Research Council dyspnea grade: 1 / 2 / 3 / 4 / 5	14.2% / 30.3% / 30.5% / 16.2% / 6.1%
At least one exacerbation during the last 12 months	86.3%
Number of exacerbations per year	
-Mild / moderate (oral corticosteroids or antibiotics)	1.4 ± 2.3 / 1.6 ± 1.7
-Severe (hospitalization) / very severe (intensive care unit)	0.3 ± 0.8 / 0.0 ± 0.2
Comorbidities	
-CHD / CHF / RVF	14.9% / 6.5% / 7.2%
-Asthma / bronchiectasis / sleep apnea syndrome	11.9% / 6.3% / 8.6%
-Depression	7.9%
Investigations and follow-up during the past 12 months	
-Arterial blood gases / EKG	70.0% / 68.8%
-CT-scan / echocardiography	61.6% / 50.6%
-Sleep oximetry / polysomnography	23.2% / 15.4%
-6MWT	27.9%
Visits to respiratory physicians / GPs	2.6 ± 1.7 / 6.7 ± 4.1
Treatments	
-ICS / Fixed ICS + LABA combinations	22.8% / 51.5%
-SABA / SABA + SAMA	34.9% / 29.9%
-LABA / LAMA	26.6% / 17.0%
-Theophylline / oral corticosteroids	8.3% / 4.5%
-LTOT / LTNIV	16.6% / 4.8%
-Influenza / pneumococcal vaccines	81.2% / 58.2%
-Rehabilitation during the last 2 years	26.9%

CHD: coronary heart disease; CHF: congestive heart failure; RVF: right ventricular failure; EKG: electrocardiogram; 6MWT: 6-minutes walk test; GP: general practitioner; ICS: inhaled corticosteroids; SABA/LABA: short/long-acting beta-agonists; SAMA/LAMA: short/long-acting anticholinergics; LTOT/LTNIV: long-term oxygen therapy / non-invasive ventilation.

### Definition of clinical subtypes by MCA, clustering and their combination

Detailed results of MCA and cluster analysis of clinical subtypes are provided in the electronic supplementary material. Altogether, MCA allowed defining 9 groups within the first four interpretable axes, while 7 clusters were identified from clustering. Putting all these results together lead to define 6 clinical subtypes (Table 2). It has to be noted that these subtypes are not exclusive: for instance, a given patient may belong to subtype 4 (women) and subtype 1 (mild severity).

**Table 2:** **Clinical subtypes identified by combination of multiple component and clustering analyses**, and their relations with treatment subgroups

**Clinical subtypes**	**Description, frequency and associated treatment type**
1 : Exposed but not severely impaired patients	Exposed to tobacco smoke or occupational smokes, toxic gaz or dust. No severe airflow obstruction (VEMS > 60%). MRC grade 0 (maximum MRC grade = 2).
	9.62%* of patients met the first three conditions.
	Patients of this clinical subtype are underrepresented in all treatment subgroups and particularly respiratory support (treatment group 4: OR = 0.198). All treatment types are less prescribed to this clinical subtypes and particularly respiratory support (treatment subgroup 4: OR = 0.255).
2 : Overweight smokers with high blood pressure and other comorbidities	Sleep apnea. Men. Robust stature (weight >80 kg). Current smokers. Underwent polysomnography. High blood pressure. Large variety of other comorbidities. Rather mild or moderate MRC grade.
	6.50%^*^ of patients met the first three conditions.
	Patients of this clinical subtype are overrepresented among prescriptions of respiratory support (treatment subgroup 4: OR = 3.625) and underrepresented among prescriptions of vaccines (treatment subgroup 6: OR = 0.581).
3 : Severe airflow obstruction	Severe dyspnea (MRC grade 4 or 5). Low FEV_1_ (< 50%) and FVC. Chronic right ventricular failure. Emphysema. Many investigations performed or prescribed including lung CT-scan, bronchoscopy, DLCO, 6-min walking test, sleep oxymetry, arterial blood gases, exercise testing, echocardiography, EKG, lung scintigraphy.
	16.06%* of patients met the first two conditions.
	Patients of this clinical subtype are overrepresented in all treatment subgroups and particularly respiratory support (treatment subgroup 4: OR = 6.214). All treatment subgroups are more prescribed to this clinical subtype, and particularly respiratory support (treatment subgroup 4: OR = 7.381).
4 : Women	Women. Small stature (< 66 kg, < 165 cm). Small respiratory capacity (FEV_1_ < 1 l, FVC < 2 l). 60 years old or more. MRC grade can be severe. Often living alone. Possibly depressive. Living in town. Associated asthma is possible. Were prescribed reversibility testing.
	12.70% ^*^of patients met the first three conditions.
	Patients of this clinical subtype are not significantly overrepresented or underrepresented in any treatment subgroup and no treatment subgroup is significantly more (or less) prescribed to this clinical subtype (no special treatment for women).
5 : Patients with symptoms of chronic bronchitis	Chronic cough and sputum production, chronic bronchitis. Fibrous or cavity sequelae and bronchiectasis in some patients. Not strongly related to MRC grade but rather moderate.
	67.07%^*^ of patients met the first three conditions.
	Patients of this clinical subtype are not significantly overrepresented or underrepresented in any treatment subgroup except for vaccines (type 6: OR = 2.210) and no treatment subgroup except for type 6 is significantly more (or less) prescribed to this clinical subtype..
6 : Elderly patients with cardiovascular comorbidity	Age > 75 years. Heart diseases: heart failure, ischemic heart disease or other cardiovascular diseases.Not strongly related to MRC grade but rather moderate. High blood pressure. Possible peripheral artery disease and cancer.
	11.06%^*^ of patients met the first and at least one of the 3 other conditions.
	Patients of this clinical subtype are significantly underrepresented in nebulised treatment (treatment subgroup 1: OR = 0.686) and overrepresented in fixed combinations (treatment subgroup 2: OR = 1.335) and vaccines (treatment subgroup 6: OR = 1.335). Nebulised treatments are significantly less prescribed to this clinical subtype. Fixed combinations and vaccines are significantly more prescribed to these patients.

Associated treatment subgroups (see Table 3) are those obtained through logistic regressions (see Table 4).

* The sum of percentages is larger than 100% because clinical subtypes are not exclusive. For example the same patient may belong to subtype 1 and 6.

### Treatment subgroups defined by MCA, clustering and their combination

Detailed results of MCA and cluster analysis of treatment characteristics are provided in the electronic supplementary material. Briefly, again four axes of the correspondence analysis were interpretable and MCA allowed defining 5 groups while clustering found 6 clusters. The combination of ordination and clustering lead to distinguish 6 treatment subgroups (Table 3). Oral corticosteroids belong to treatment subgroup 1 (nebulised treatments) because they can be associated with (i.e., they are not negatively related to) nebulised drugs. MCA and clustering show that treatments subgroups 1 and 2 (fixed ICS + LABA combinations) are usually mutually exclusive (opposite position on axis 1, and linkage at a negative correlation). Like clinical subtypes, treatment subgroups are not exclusive and two types of treatments may therefore be prescribed to the same patient.

**Table 3:** Treatment subtypes identified by combination of multiple component and clustering analyses

**Treatment subgroups**	**Description, frequency**
1: Nebulised treatments	Nebulised anticholinergics and/or beta2 agonists. Less frequently, nebulised corticosteroids.
	One of the first two treatments was prescribed to 45.55% of patients.
2: Fixed combinations	Combinations of long acting beta 2 agonist and corticosteroids.
	These combinations were prescribed to 51.44% of patients.
3 : LABA and inhaled corticosteroids prescribed separately	LABA and inhaled corticosteroids, co-prescribed in 91.2% of patients who receive one and/or the other outside of a fixed combination.
	Such “separate associations” were prescribed to 16.03% of patients.
4: Non-invasive ventilation and oxygen therapy	Ventilation or oxygen therapy. Respiratory support may be prescribed with respiratory rehabilitation.
	One of the first two therapies was prescribed to 18.71% of patients.
5: Fixed combinations of short-acting anticholinergic and β2 agonist	These treatments are not prescribed in patients who receive long acting anticholinergics. Prescription of a fixed combination of LABA and ICS is possible and independent (neither association nor exclusion).
	These fixed combinations were prescribed to 29.85% of patients.
6: Flu and pneumococcal vaccines	Flu or pneumococcal vaccines and antibiotics, sometimes associated with chest physiotherapy.
	At least one of the two first vaccines and antibiotics were prescribed to 39.83% of patients.

### Relationship between clinical subtypes and treatment subgroups

Odds ratio (OR) estimated by the 12 multivariate logistic regressions are presented in Table 4. Each row indicates whether the clinical subtype is overrepresented (OR > 1) or under-represented (OR < 1) in the prescriptions of each treatment type. Each column indicates if the treatment type is preferably prescribed (OR > 1) or preferably avoided (OR < 1) in patients of a given clinical subtype. Odds ratios within the same cell of the table are slightly different because they are not adjusted on the same set of covariates (see the methods section). Overall, the results show that associations are more quantitative than qualitative in that all treatment types are significantly less prescribed to less severe patients (i.e., patients subtype 1) and more prescribed to the most severe (patients subtype 3). More specific associations were as follows: fixed combinations (treatment subgroup 2) are significantly more prescribed to elderly patients (patients subtype 6) while nebulized treatments (treatment subgroup 1) are significantly less prescribed to these patients. Vaccines (treatment subgroup 6) are more prescribed in patients with symptoms of chronic bronchitis (subtype 5) and respiratory support (treatment subgroup 4) is significantly related to clinical subtype 2 (overweight smokers with comorbidities).

**Table 4:** Multivariate logistic regressions: Odds ratio for associations between each clinical subtype and treatment subgroups (first line of each cell in a row) and each treatment subgroup and clinical subtypes (second line of each cell in a column, in italic)

**Odds ratio**	***Trt type 1***	***Trt type 2***	***Trt type 3***	***Trt type 40***	***Trt type 5***	***Trt type 6***
	**Nebulised treatments**	**Fixed combinations**	**LABA and inhaled corticosteroids prescribed separately**	**Non-invasive ventilation and oxygen therapy**	**Fixed combinations of short acting anticholinergic and β2 agonist**	**Flu and pneumococcal vaccines**
Clinical type 1						
Exposed but not severely impaired patients	**0.611**^3^	**0.713**^1^	**0.581**^1^	**0.198**^4^	**0.592**^2^	**0.575**^3^
	***0.636***^*2*^	*0.800*^*0*^	***0.686***^*1*^	***0.255***^*3*^	***0.637***^*2*^	***0.500***^*4*^
Clinical type 2						
Overweight smokers with comorbidities	0.781^0^	0.907^0^	0.891^0^	**3.625**^4^	1.060^0^	**0.581**^2^
	*0.877*^*0*^	*0.939*^*0*^	*1.004*^*0*^	***4.396***^*4*^	*1.221*^*0*^	*0.762*^*0*^
Clinical type 3						
Severe airflow obstruction	**1.636**^3^	**1.605**^3^	**1.580**^1^	**6.214**^4^	**1.382**^1^	**1.606**^3^
	***1.683***^*4*^	***1.455***^*3*^	*1.207*^*0*^	***7.381***^*4*^	***1.381***^*2*^	***2.095***^*4*^
Clinical type 4						
Women	1.142^0^	0.952^0^	1.293^0^	0.821^0^	1.250^0^	1.105^0^
	*1.097*^*0*^	*0.868*^*0*^	*1.274*^*0*^	*1.101*^*0*^	*1.254*^*0*^	*1.194*^*0*^
Clinical type 5						
Symptoms of chronic bronchitis	1.116^0^	0.986^0^	0.868^0^	1.117^0^	1.095^0^	**2.210**^4^
	*1.161*^*0*^	*1.083*^*0*^	*0.912*^*0*^	*1.243*^*0*^	*1.107*^*0*^	***2.221***^*4*^
Clinical type 6						
Elderly patients with cardiovascular comorbidity	**0.686**^2^	**1.335**^1^	1.022^0^	1.308^0^	0.954^0^	**1.441**^2^
	***0.733***^*1*^	***1.323***^*1*^	*0.899*^*0*^	*1.200*^*0*^	*1.087*^*0*^	***1.351***^*1*^

All models included the 6 treatment or clinical subtypes as covariates. Figures in bold are significantly different from 1 and exponent indicates the class of p values of the log-likelihood test: ^0^ for p > 0.05, ^1^ for p ≤ 0.05, ^2^ for p < 0.01, ^3^ for p < 0.001 and ^4^ for p < 0.0001.

### Overall relationships between patients’ characteristics and pharmacological and non-pharmacological treatments

Only 6.23% of variations in the use of pharmacological treatments are explained by patient characteristics as described by 6 families of variables: Table 5 shows the components of each of these families and the percentage of treatment variations that it explained. Altogether, the described associations are vague and reflect trends or tendencies rather than tight relations. Significant explanatory variables for components of care other than medications are shown in Table 6. Altogether, 14.29% of variations of these components of care are explained by patient characteristics.

**Table 5:** Explanation of the overall treatment variation by 6 families of clinical variables

**Family**	**Selected variables**	**Adjusted R**^**2**^	**P value**
Socio-demographics	stature, age, lifestyle (living alone,…),	0.0047	0.001
History of COPD and comorbidities	history of right ventricular failure, emphysema, sleep apnea, asthma, dyspnea, chronic sputum production, ischemic heart disease.	0.01998	0.001
Symptoms the days of visit	dyspnea (MRC), chronic bronchitis	0.02515	0.001
Lung function	FEV_1_, FEV_1_%, FVC,	0.02120	0.001
Exacerbations and smoking status	number of exacerbations, current smoking, current occupational exposure to dust or smoke	0.00626	0.00494
Investigations (d. stands for done and p. for prescribed)	sleep oxymetry d., walking test d., arterial blood gases d., scintigraphy d., polysomnography d., ECG d., VO^2^max d., VO^2^max p., 6MWT p., DLCO p., scintigraphy p., EKG p.	0.03927	0.001

The order of variables is the order of their contribution to the explanation of the variation of COPD treatments. *d*.: done; *p*.: prescribed.

**Table 6:** Explanation of overall variation in non-pharmacological care (other treatments and follow up) by 6 families of variables

**Family**	**Selected variables**	**Adjusted R**^**2**^	**P value**
Socio-demographics	age, weight, town, gender	0.02517	0.001
History of COPD and comorbidities	emphysema, chronic sputum production, dyspnea, history of right ventricular failure, bronchiectasis, ischemic heart disease, asthma, cough, depression, heart failure.	0.04009	0.001
Symptoms the days of visit	dyspnea (MRC), chronic bronchitis	0.05125	0.001
Lung function	FEV_1_, FEV_1_%, reversibility testing, FVC	0.03641	0.001
Exacerbations and smoking status	number of exacerbations, current smoking, current occupational exposure to dust or smoke	0.04385	0.001
Investigations (d. stands for done and p. for prescribed)	6MWT d., CT-scan d., echocardiography d., arterial blood gases d., scintigraphy. d., nocturnal oxymetry d., 6MWT p., arterial blood gases p., EKG d., DLCO p., fiberoptic bronchoscopy d., DLCO d., EKG p.	0.07245	0.001

The order of variables is the order of their contribution to the explanationof the variation of COPD treatments.

## Discussion

In this large sample of COPD patients cared for by respiratory physicians, several approaches to factorial analysis were used in a step by step manner to identify associations between administered treatments on the one hand, and clinical subtypes on the other. Firstly, multiple correspondence and cluster analyses allowed identification of sufficiently robust clinical and treatment subgroups. Then canonical analysis of redundancy showed that the fraction of variation in pharmacological treatments explained by patient characteristics was small (6.23%) and half that observed for non-pharmacological treatments. This approach appears useful to identify areas of uncertainty in prescription choices by physicians.

### Strengths and limitations of the study

Using factor analyses was justified primarily by (i) the large number of patients characteristics and possible therapies as well as (ii) the known wide overlap between GOLD stages of airflow obstruction for most clinical variables used to describe patients (such as dyspnea or exacerbation frequency), which makes it difficult to identify subtypes using conventional analysis. Factor analysis is basically a descriptive approach that summarizes associations without any *a priori* hypothesis. Indeed, it confirmed that many factors other than FEV_1_ add to the description of the population: risk factors, symptoms (chronic bronchitis, dyspnea), comorbidities, demographics (age, gender) and anthropometric characteristics (weight) are also important contributors to characterization. The main limitation of these methods is their high dependency on active variables that participate to the construction of axes. Addition or subtraction of some variables (characteristics) may change the typology. Moreover, the choice of characteristics constituting one group (subtype) is not fully objective (preferential collection of characteristics that are believed to be relevant). Thus, the choice of recorded variables may be questioned: some variables that may predict treatment choices may have been omitted when building the case-report form; this could participate to explain why clinical characteristics as a whole were poorly associated to treatment subgroups. However, it must be outlined that the selection of collected data included all patients and disease characteristics that are routinely accessible to any practitioner: therefore, if some additional information was lost (*e.g.,* degree of emphysema on CT-scan, lung volumes or diffusion capacity, characteristics of airway inflammation as measured in induced sputum), it would not be relevant in routine clinical practice.

The large sample size of the present study provides a good power for detecting associations (80% chance of detecting a true correlation of 0.057 and 70% chance of detecting a true correlation of 0.05) and therefore allows considering that, if some real relations are not detected, they would be very small and of no clinical relevance. However, it might also be that treatment choices rely on factors completely different from those recorded in our study. We believe that this last explanation is rather unlikely since virtually all routinely collected clinical and lung function characteristics of COPD were assessed.

Another limitation to be addressed relates to the statistical methods used for factorial analyses: a group of redundant characteristics (information) may be well represented because of their association (redundancy) while an independent clinically relevant information could be lost in the blank noise and not be well represented by the interpretable axes. The risk of such an event is minimized by the use of 3 different statistical approaches that search for associations through different procedures. For example, cluster analysis does not lose any variable in the blank noise.

We did not include the new GOLD classification (A-B-C-D) [1] into the analyses, Indeed, our purpose was to study the relationship between treatment decisions and patients’ characteristics with no a priori hypothesis rather than to test the relations between treatments and already defined patients subgroups such as those proposed in the new GOLD document. Interestingly, the variables that participate most to the definition of the patients subgroups identified by cluster analyses actually do not fit with those used in the GOLD A-B-C-D classification (see Table 2). Therefore, this classification is quite unlikely to by closely related to treatment choices. Finally, the new GOLD classification was not available when the study was performed, and could therefore not be used *per se* for treatment choices by physicians.

While care for COPD is quite heterogeneous in Europe, the present study was performed in only one European country where local guidelines (taking into account the GOLD documents) exist and have been widely disseminated, Therefore, it might have been hypothesized that practice would be more homogeneous than in a multinational survey. Even though, it remained difficult to determine how physicians make their choices.

### Associations between treatments and clinical characteristics

There appeared to be separate gradients in the use of ICS + LABA, short acting bronchodilators, nebulised treatments and oral corticosteroids, and respiratory support. Such data suggest that several independent (at least in part) factors influence treatment choices. The very low variation of pharmacological treatments explained by clinical subtype is in line with data from other studies, which showed that the treatment of COPD is notably heterogeneous, either for stable disease or exacerbations. This can be due to several factors. Firstly, some physicians may have their own “specific” and “patient-independent” therapeutic habits. This could have prevented us from detecting an overall strategy since the investigator effect could not be taken into account due to the too low number of patients per physician. Secondly, the investigator’s therapeutic strategy may depend mostly on what was already prescribed to the patient in the past. This might not be detected since this was a cross-sectional study, providing only a snapshot of the situation at a given time and preventing from capturing the evolution of the treatment strategy over time. In addition, we did not record treatment changes that were decided during the visit, which would have allowed us to detect how new prescriptions were influenced by previous treatments. Thirdly and most importantly, physicians may consider that currently available treatments are not markedly different in terms of efficacy and safety profile. This would be in accordance with the impossibility to identify well-defined subgroups of responders or non-responders in recent major treatment trials [16-20]. This can be explained in two ways: either recorded patients characteristics are not the correct predictors of response, or response is not adequately defined (e.g., by FEV_1_, which is used in most studies). Indeed, available guidelines are quite vague about clinical criteria that may allow to choose, *e.g.,* between available classes of inhaled bronchodilators. This relates to the lack of convincing evidence on the superiority of any treatment in well-defined subgroups, even in large randomised controlled trials [16,17]. Considering these observations, there is a clear need for further research on phenotypes and predictors of response to treatments.

Even though, efforts should also be directed at improving adherence of physicians to guidelines and understanding the determinants of their therapeutic choices. Dissemination is obviously required but also clearly insufficient to ensure wide guidelines implementation and appropriate treatment use. Barriers have to be identified and may relate to guidelines themselves (when unclear or too difficult to implement) but also to physicians (inertia,lack of awareness, familiarity, agreement, self-efficacy, outcome expectancy, ease of prescription, anticipated drawbacks), patients (underlying the importance of taking their preferences into account) or healthcare systems (“external barriers”). These aspects have been extensively reviewed elsewhere, especially in a 2005 Canadian Wokshop focused on asthma and COPD [28].

However, it must also be underlined that explaining 6.23% of treatment variations by patients characteristics is not amazingly low compared to results obtained in other therapeutic areas: in most studies of the same kind with other medications available on the market (*e.g.,* anti-depressants), less than 10% of treatment choices can be attributed to specific patients characteristics. Thus, even if the percentage of explained variation is not large, relationships were found and the two main criteria for the choice of treatments according to current guidelines, FEV_1_ and exacerbations, were confirmed as predictors of treatment choices in MCA and canonical analyses. Moreover, it was possible to go further and find far more associations with other factors including dyspnea and comorbidities. Finally, it must be kept in mind that individual treatment responses were not recorded in the study. Therefore, our findings do not refute the hypothesis of specific phenotypes based on response to treatments. Actually, we cannot test this hypothesis due to the cross-sectional nature of the study. While we can conclude that the choice of a given treatment does not markedly relate to patients’ characteristics at a given time-point, we cannot exclude that it relates to improvements reported by the patient or recorded by the physician, following the first prescription of this treatment.

The % of variation of non pharmacological treatments that is explained by clinical characteristics of patients represents more than twice the amount of variation of medications for COPD that was explained by these characteristics. Components of care other than medications are thus more dependent upon patient characteristics or/and more stereotyped across physicians than medication strategy, which may suggest greater confidence in the specific (or differentiated) effects of non-pharmacological treatments. Moreover, clinical variables explaining non-pharmacological treatments are not strictly the same as for medications, which reflects the use of different patient characteristics for adjusting different components of care.

## Conclusions

In conclusion, it is clear from this study that, at present, patients’ characteristics are not an overwhelming factor for explaining the therapeutic strategy in stable COPD.This suggests the need to rationalize treatment choices. To achieve this, it would be useful to increase our knowledge on clinical subtypes and their association with treatment responses. Such information could be obtained from factor analyses applied on large databases, observational studies and large long-term therapeutic trials: this type of analyses appears capable of identifying patients and treatments subtypes and analysing their relationship. Altogether, the methods used here allow identification of areas of uncertainties in prescriptions and may provide opportunities to identify responders both in clinical trials and in the real life.

## Abbreviations

6MWT: 6-minutes walk test; AHCLCA: Ascending hierarchical complete linkage clustering analysis; CAC: Canonical analysis of correspondence; CHF: Congestive heart failure; COPD: Chronic obstructive pulmonary disease; EKG: Electrocardiogram; FEV_1_: Forced expiratory volume in one second; FVC: Forced vital capacity; GOLD: Global initiative for obstructive lung disease; GP: General practitioner; ICS: Inhaled corticosteroid; LABA: Long-acting beta2 agonist; LAMA: Long-acting antimuscarinic agent; LTNIV: Long-term non-invasive ventilation; LTOT: Long-term oxygen therapy; MCA: Multiple correspondence analysis; MRC: Medical research council dyspnea scale/grade; OR: Odds ratio; RVF: Right ventricular failure; SABA: Short-acting beta2 agonist.

## Competing interests

NR received support for attending scientific meetings and fees for speaking, organizing research or consulting from Almirall, AstraZeneca, Boehringer Ingelheim, Chiesi, GlaxoSmithKline, Hoffman la Roche, Mundipharma, MEDA, Novartis, Nycomed/Altana, Pfizer, TEVA.

HP received support for attending scientific meetings and fees for speaking, consulting from Astra-Zeneca, Boehringer Ingelheim, Chiesi, GlaxoSmithKline, Novartis, Pfizer.

JMM received support for attending scientific meetings from Almirall, AstraZeneca, Boehringer Ingelheim, Chiesi, GlaxoSmithKline, MSD, Novartis, Pfizer.

YM received support for attending scientific meetings and fees for speaking, consulting or research from AstraZeneca, Boehringer Ingelheim, Chiesi, Nycomed/Altana.

BD: no competing interest.

CC received support for attending scientific meetings and fees for speaking, organizing research, or consulting from AstraZeneca, Boehringer Ingelheim, GlaxoSmithKline, Hoffman la Roche, Lilly and Amgen.

## Authors’ contributions

All authors contributed to study design and follow-up, manuscript writing and validation of the final version. NR and BS wrote manuscript drafts. BS coordinated statistical analyses.

## Pre-publication history

The pre-publication history for this paper can be accessed here:

http://www.biomedcentral.com/1471-2466/12/39/prepub
